# Hospital Administration

**Published:** 1896-07-04

**Authors:** 


### HOSPITAL ADMINISTRATION.

### PROVISION FOR INCURABLES.

A fact which does not seem to be generally known is that several of the London voluntary hospitals, besides ministering to those patients whose ailments it is possible, with skill and attention, to cure, also make provision for cases which are practically incurable. Thus the Middlesex Hospital devotes large sums of money each year to the maintenance of its wards for cancer patients; the Westminster Hospital has an "Incurables Fund"; a small allowance is made by the North London Hospital for Consumption to the most deserving cases discharged as incurable; the National Hospital for the Paralysed and Epileptic has a pension fund for incurable cases caused by paralysis or epilepsy; and so on. We do not think it will be out of place if we briefly describe the system on which the Pension Fund at the National Hospital for the Paralysed and Epileptic is worked, seeing that it is one of the largest of the funds which are attached to hospitals for this purpose. Altogether at the present time there are 88 pensions, ranging from £22 10s. to £10 a year, to which applicants are elected by the vote of the governors of and subscribers to the hospital. Fortunately when first establishing these pensions the founders were alive to the great abuses which attach to the ordinary modes of election at most of the pension-giving institutions, and in consequence decreed that not only should canvassing in any way or buying of votes be prohibited, but that such proceedings should disqualify the candidates for election. The candidates are, therefore, saved the great trouble and expense which they are put to under the ordinary voting system of election, and the voters are left to their individual discretion as to which is the most deserving of the candidates submitted by the hospital authorities. As may be well imagined, the 88 pensions now in existence cannot nearly provide for the many deserving incurable cases suffering from the diseases treated at the National Hospital for the Paralysed and Epileptic. It will, therefore, be useful to let our wealthy readers know what they can do towards remedying this. A pension of £10 a-year may be founded in perpetuity for the sum of £300, one of £15 a-year for £450, and so on, whilst a donation of £120 will found a pension of £10 a-year during the life of the donor. The nomination to a particular pension may if so desired rest in the founder during his lifetime. In addition to this the board will increase any one of the existing pensions by £5 annually during the life of the present holder on receipt of a donation of £60 to the pension fund. We need not say more, as we feel sure it is only necessary to make more widely known the fact that such an excellent work is being done, to ensure that its necessities will be ministered to by those who have funds for charitable purposes at their disposal.

